# Hand Infections Across Tissue Planes: A Pictorial Review

**DOI:** 10.7759/cureus.111341

**Published:** 2026-06-23

**Authors:** Una Milovanovic, Derek S Weimer, Melissa Ricardo, Drew Johnston, Gary Schwartz

**Affiliations:** 1 Medical Education, Dr. Kiran C. Patel College of Allopathic Medicine, Nova Southeastern University, Fort Lauderdale, USA

**Keywords:** bite wounds, cellulitis, deep space infections, flexor tenosynovitis, hand infections, onychomycosis, osteomyelitis, paronychia, pictorial review, septic arthritis

## Abstract

Infections of the hand are encountered across emergency medicine, primary care, and surgical practice, and their outcomes depend heavily on early recognition. The complex anatomy of the hand, with its multiple fascial planes, tendon sheaths, and confined spaces, allows infection to spread rapidly and produce lasting functional impairment when treatment is delayed. This pictorial review presents a series of clinically important hand infections drawn from the archive of a board-certified hand surgeon, spanning common superficial conditions to uncommon but limb-threatening deep-space, articular, and osseous disease. For each entity, we summarize characteristic clinical features, useful diagnostic and imaging findings, likely causative organisms, and initial management, illustrated with representative clinical photographs and imaging. By organizing these conditions across tissue planes, the review offers a practical, pattern-based reference that clinicians can use at the point of care to triage urgency, select empiric therapy, and identify patients who require early referral or operative intervention.

## Introduction and background

Infections of the hand are encountered across emergency medicine, primary care, and surgical practice [1,2]. The complex anatomy of the hand, characterized by multiple fascial planes, tendon sheaths, and confined spaces, allows infection to spread rapidly and cause significant functional impairment if not promptly addressed [3]. Clinicians must be able to distinguish superficial infections from deeper or compartmental infections that require urgency [4,5].

This pictorial review provides an overview of clinically important hand infections ranging from common superficial conditions to uncommon but severe deep-space and systemic infections, with emphasis on their clinical features, diagnostic considerations, and management strategies. By integrating clinical photographs and imaging, this review aims to serve as a practical reference for healthcare providers in recognizing infection patterns and preventing functional loss through early, evidence-based intervention.

This retrospective review evaluated 14 clinical cases of hand infections obtained from the archives of a board-certified hand surgeon. Cases were purposively selected to represent the most clinically important and educationally illustrative presentations across tissue planes, based on the availability of high-quality clinical photographs and imaging. The cases include paronychia, onychomycosis, thenar space abscess, cellulitis, septic flexor and extensor tenosynovitis, cat bite, dog bite, human bite, septic arthritis of the distal interphalangeal joint, septic arthritis of the wrist, osteomyelitis of the distal phalanx, and diabetic hand gangrene. All materials were de-identified prior to inclusion. This study was conducted for educational purposes only and did not involve identifiable patient information. Informed consent for treatment and open access publication was obtained or waived by all participants in this study. Institutional review board (IRB) approval was not required, nor was it obtained.

## Review

Superficial and periungual infections

These are the most common infections seen in the primary care setting and often present early.

Paronychia

**Figure 1:**
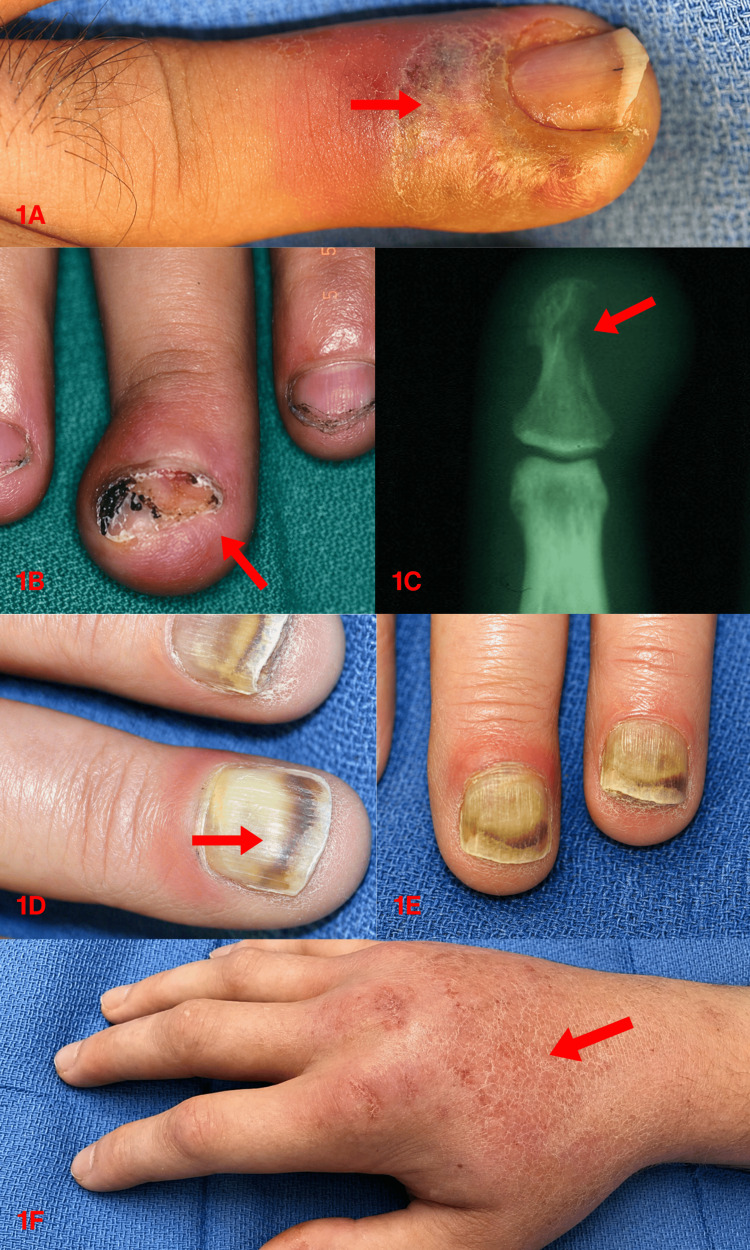
Superficial and periungual infections (A) Gross examination of acute paronychia with early abscess formation, showing localized erythema and fluctuant swelling of the proximal nail fold following trauma to the cuticle (red arrow). (B-C) A case of presumed chronic paronychia with nail dystrophy: gross examination (B) shows a thickened nail plate with periungual erythema and nail dystrophy (red arrow); radiograph (C) shows soft tissue fullness and nail matrix distortion (red arrow). The lesion was unresponsive to treatment, and biopsy confirmed subungual squamous cell carcinoma, highlighting the importance of considering malignancy in refractory nail conditions [7]. (D-E) Onychomycosis with subungual hyperkeratosis, distal onycholysis, and yellow-brown nail discoloration (red arrow) involving multiple fingernails, consistent with chronic dermatophyte infection. (F) Acute cellulitis with diffuse dorsal hand erythema and swelling with ill-defined borders (red arrow). Image courtesy of Gary Schwartz, MD. Used with permission.

Paronychia is a superficial infection of the nail folds, commonly the lateral and proximal surfaces, and is among the most common hand infections [1,6]. Acute paronychia presents within six weeks of onset, usually after disruption of the nail fold barrier from nail biting, hangnail picking, or artificial nails. *Staphylococcus aureus* is the most frequent pathogen, followed by *Streptococcus pyogenes*, *Pseudomonas*, and anaerobes. Patients develop localized tenderness, erythema, and swelling that may progress to abscess formation (Figure 1A). Management includes warm soaks, topical antibiotics such as mupirocin, and, if severe, oral antibiotics such as clindamycin or amoxicillin-clavulanate. To avoid persistent infection, incision and drainage are required for abscesses [4]. Chronic paronychia, though similar in presentation to the acute form, lasts longer than six weeks and is often due to chronic moisture exposure or irritants. *Candida albicans* is most commonly isolated. Management involves avoiding irritants, restoring the nail barrier, and applying topical corticosteroids or tacrolimus [4,6]. Persistent or treatment-refractory periungual lesions warrant biopsy to exclude malignancy (Figure 1B,C).

Onychomycosis

Onychomycosis is a chronic fungal infection of the nail plate and bed, most commonly caused by *Trichophyton rubrum* or *Trichophyton mentagrophytes* [8]. Risk factors include advanced age, diabetes mellitus (DM), peripheral vascular disease, tinea pedis, and repeated nail trauma [9,10]. Patients typically present with nail thickening, discoloration (yellow, brown, or white), and progressive brittleness (Figure 1D,E). Although diagnosis is often clinical, confirmation with potassium hydroxide preparation, fungal culture, or periodic acid-Schiff staining of nail clippings is recommended to exclude mimickers such as psoriasis or lichen planus [11]. Oral terbinafine 250 mg daily or itraconazole 200 mg daily for six to 12 weeks are first-line therapies. Topical agents such as efinaconazole 10% or ciclopirox 8% may be used for mild or limited disease. Nail care, treatment of coexisting tinea pedis, and patient adherence are essential to prevent recurrence.

Cellulitis

Cellulitis is an acute bacterial infection of the dermis and subcutaneous tissue that occurs when bacteria enter through a compromised skin barrier [5,12]. The most common pathogens are *Streptococcus pyogenes *and *Staphylococcus aureus *[13,14]. Animal bites may introduce *Pasteurella multocida*, while human fight bite injuries over the dorsal metacarpophalangeal joint can extend to deeper structures, causing septic arthritis or osteomyelitis, often involving *Eikenella corrodens *[15]. Clinical presentation includes erythema, swelling, warmth, and tenderness with indistinct borders (Figure 1F); severe cases may show lymphangitic streaking or purulent drainage [5]. Though diagnosis is primarily clinical, imaging is indicated when an abscess or deep infection is suspected. Empiric antibiotics should cover *Streptococcus* and *Staphylococcus*, with oral cephalexin as first-line therapy; coverage for methicillin-resistant *Staphylococcus aureus *(MRSA) should be added in cases of purulent cellulitis, failed outpatient therapy, or known risk factors [14,16]. Early treatment prevents abscess formation, osteomyelitis, and systemic spread [14].

Closed-space and deep compartment infections

These are surgical emergencies and highlight the importance of rapid referral and multidisciplinary care.

Closed-Space Infections

**Figure 2:**
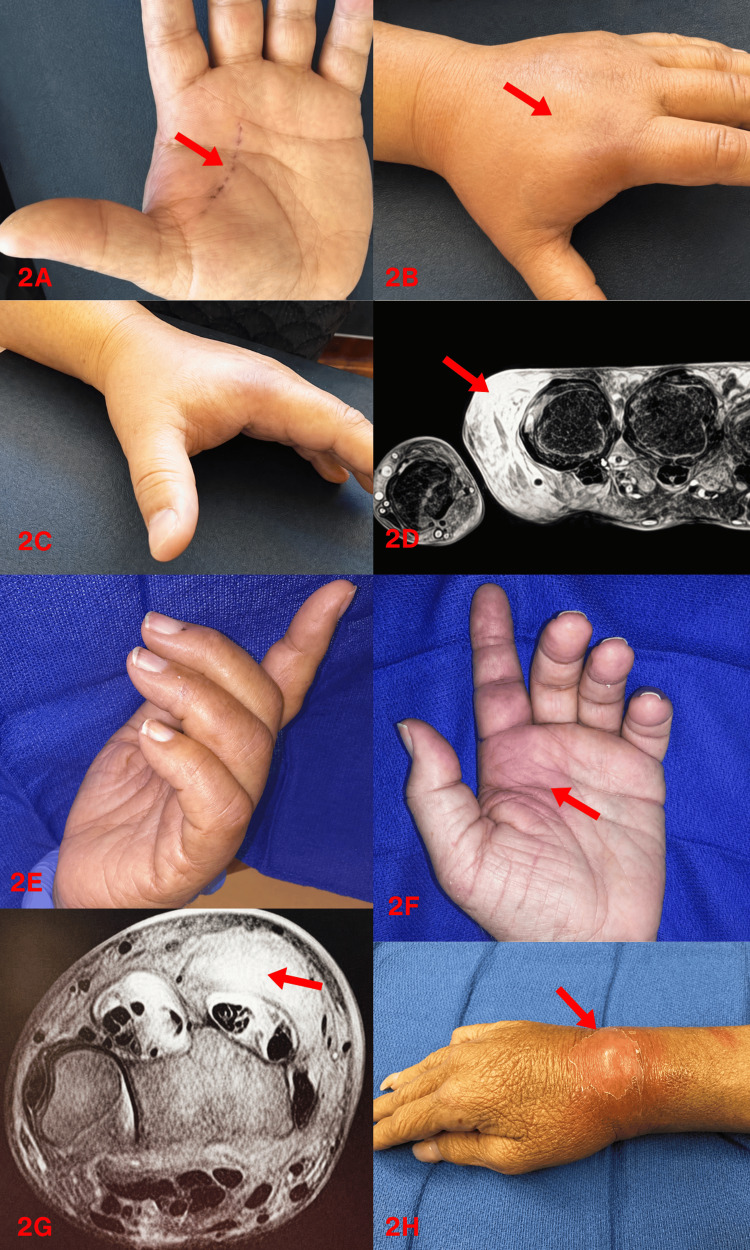
Closed-space and deep compartment infections (A-D) Closed space infection: gross examination (A-C) shows a puncture wound in the thenar region (red arrow, A) with surrounding swelling and erythema (red arrow, B); axial MRI (D) shows a localized fluid collection with surrounding edema (red arrow), indicating abscess in the thenar compartment. (E-F) Septic flexor tenosynovitis with fusiform digit swelling in a flexed posture, erythema (red arrow, F), and tenderness along the flexor tendon sheath. (G-H) Extensor tenosynovitis: axial MRI (G) demonstrates fluid accumulation and synovial thickening (red arrow) in the second and fourth dorsal compartments; gross examination (H) shows marked erythema and swelling over the dorsal wrist with overlying skin tautness (red arrow). Image courtesy of Gary Schwartz, MD. Used with permission. MRI: magnetic resonance imaging.

Closed space infections of the hand involve the tendon sheaths, deep fascial compartments, and joints. The three major deep palmar spaces, including the thenar, hypothenar, and midpalmar spaces, each present with distinct clinical features. Thenar space infections typically cause swelling and tenderness of the thenar eminence with the thumb held in abduction and pain with thumb motion. Midpalmar space infections present with loss of the normal palmar concavity and limited motion of the middle and ring fingers. Hypothenar space infections are less common and present with localized pain and swelling over the hypothenar eminence. All three require prompt intravenous antibiotics and surgical drainage, though thenar and midpalmar space infections carry a higher risk of functional impairment given their proximity to critical tendon and neurovascular structures. Across all closed-space infections, *Staphylococcus aureus* and *Streptococcus pyogenes* are the most common pathogens, typically introduced via puncture wounds or lacerations or spread from nearby soft-tissue infections [2,17]. These infections occur most often in adults aged 20 to 50 years and are associated with diabetes, immunosuppression, intravenous drug use, or recent trauma [17]. Patients present with severe pain, diffuse swelling, erythema, warmth, and markedly limited motion (Figure 2A-D). Because the enclosed space restricts expansion, increasing pressure can cause ischemia and necrosis. Diagnosis is primarily clinical; ultrasound or magnetic resonance imaging (MRI) may help identify abscesses, while radiographs can help exclude fractures or foreign bodies [18]. First-line empiric antibiotics include amoxicillin-clavulanate or clindamycin, with urgent surgical drainage for purulent collections. Complications include tendon rupture, osteomyelitis, and permanent functional loss [12].

Septic Flexor Tenosynovitis

Septic flexor tenosynovitis is a closed-space infection of the flexor tendon sheath, accounting for up to 9% of hand infections [1]. Infection typically follows penetrating trauma, bites, or puncture wounds, most commonly from *Staphylococcus aureus *and *Streptococcus* species [19]. The four Kanavel cardinal signs (fusiform swelling, pain with passive extension, flexed finger posture, and tenderness along the flexor tendon sheath) (Figure 2E,F) are diagnostic [2]. While all four signs are central to diagnosis, not all may be present in every case, particularly in early infections. Pang et al. reported that fusiform swelling was the most consistently observed sign, present in 97% of patients, followed by pain with passive extension in 72%, semiflexed finger posture in 69%, and tenderness along the flexor tendon sheath in 64%, underscoring that a high index of clinical suspicion should be maintained even when the presentation is incomplete [20]. Imaging may show sheath distension or fluid but is not required. Immediate intravenous antibiotics covering *Staphylococcus *and *Streptococcus *(e.g., vancomycin plus piperacillin-tazobactam) and surgical irrigation and debridement are essential. Delayed treatment increases risk for tendon necrosis, stiffness, and amputation, particularly in older patients and those with DM [1].

Septic Extensor Tenosynovitis

Septic extensor tenosynovitis is an infection of the dorsal tendon sheath that typically follows penetrating trauma or bite wounds or spreads from cellulitis. It carries a similar urgency as flexor tenosynovitis because delayed treatment can lead to tendon necrosis and joint involvement. *Staphylococcus aureus *and *Streptococcus* species are the predominant pathogens, while bite-related infections may be polymicrobial, including *Pasteurella multocida* and anaerobes [21]. Patients present with dorsal hand or wrist swelling, erythema, warmth, and pain aggravated by finger extension; fluctuance or purulent drainage may be present (Figure 2H) [22]. Diagnosis is clinical, aided by ultrasound showing hypoechoic fluid and synovial thickening, or MRI demonstrating sheath and deep-space involvement (Figure 2G) [23,24]. Early empiric intravenous antibiotics such as vancomycin plus ceftriaxone or ampicillin-sulbactam for bite-related cases should be initiated promptly. Surgical irrigation and debridement are indicated for abscess, rapid progression, or lack of improvement within 24 hours [21].

Articular and osseous infections

These infections often have diagnostic delays, which puts the patient at a higher risk of long-term morbidity if missed.

Septic Arthritis of the Finger Joints

**Figure 3:**
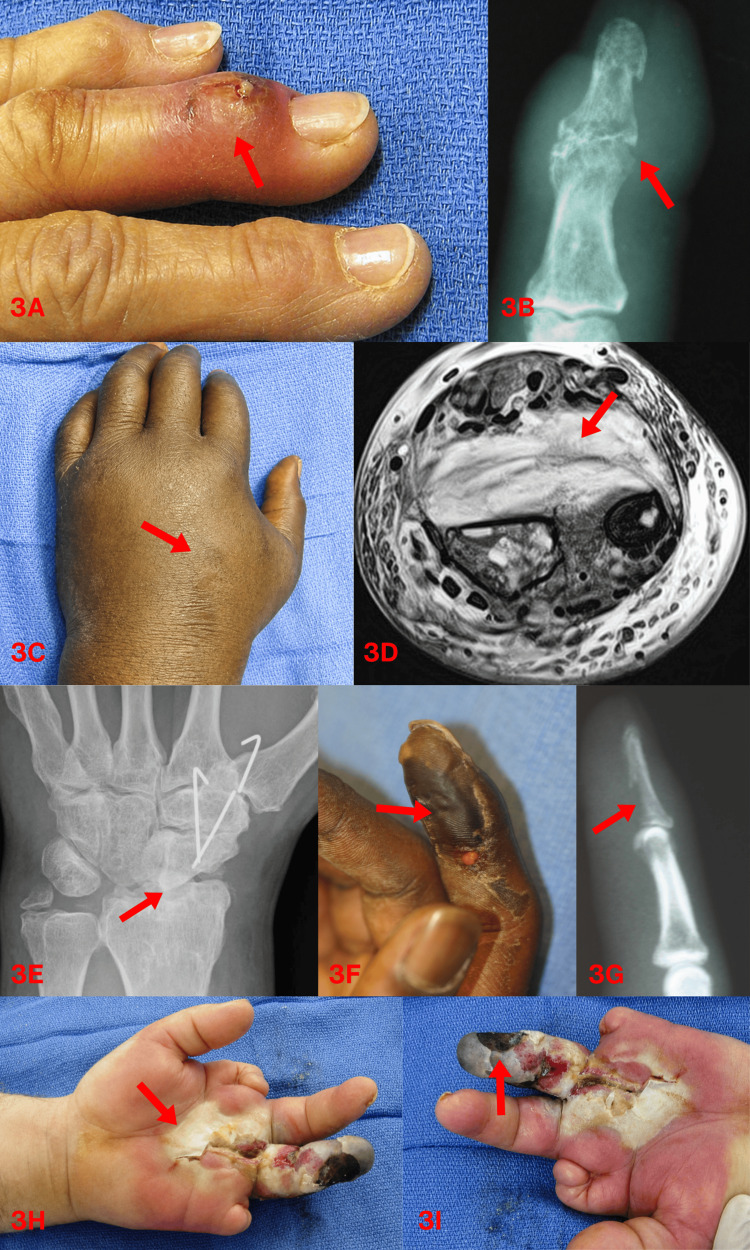
Articular and osseous infections (A-B) Septic arthritis of the finger: gross examination (A) shows swelling and erythema (red arrow) along the distal interphalangeal joint after striking a table; anteroposterior radiograph (B) shows cortical irregularity and joint space narrowing (red arrow). (C-E) Septic arthritis of the wrist: gross examination (C) shows dorsal hand swelling and erythema (red arrow) with limited range of motion; axial MRI (D) reveals joint effusion and synovial enhancement (red arrow), consistent with septic arthritis and early osteomyelitis; anteroposterior radiograph (E) shows joint space narrowing and soft tissue swelling (red arrow) of the radiocarpal joint. (F-G) Osteomyelitis of the distal phalanx: gross examination (F) shows proximal digital swelling, medial skin tension, and distal necrotic changes (red arrow); anteroposterior radiograph (G) reveals cortical erosion and bone lucency (red arrow) at the volar surface of the distal phalanx. (H-I) Gangrene secondary to uncontrolled DM, with palmar ulcerations and erosions (red arrow, H), black necrotic changes in the digits (red arrow, I), and evidence of prior surgical amputation of the index and small fingers. Image courtesy of Gary Schwartz, MD. Used with permission. DM: diabetes mellitus; MRI: magnetic resonance imaging.

Septic arthritis of the fingers is an infection within the joint space, most often involving the index and middle fingers after direct inoculation from trauma, bites, or procedures [25]. Patients present with acute joint pain, erythema, warmth, swelling, restricted motion, and a visible effusion in some cases (Figure 3A). Diagnosis requires prompt aspiration and culture of synovial fluid; ultrasound can help guide aspiration, while radiographs (Figure 3B) or, if available, MRI defines articular and periarticular involvement when needed [26]. Empiric intravenous antibiotics that cover *Staphylococcus aureus *and *Streptococcus *species should begin immediately and be tailored to culture results. Early joint drainage, irrigation, or open debridement is recommended to prevent cartilage loss and stiffness [26]. Delayed treatment, advanced age, DM, and the need for repeated procedures predict worse outcomes, including arthrosis, osteomyelitis, or amputation [27].

Septic Arthritis of the Wrist

Septic arthritis of the wrist accounts for less than 5% of all cases of septic arthritis and most often occurs in adults aged 50 to 70 years [28, 29]. It commonly results from direct inoculation via trauma, bite wounds, or invasive procedures, though hematogenous spread can occur. *Staphylococcus aureus* and *Streptococcus *species are the most frequent pathogens, while *Pseudomonas *and *Neisseria gonorrhoeae *should be considered in immunocompromised and sexually active patients, respectively [30, 31]. Patients present with acute wrist pain, erythema, warmth, swelling, and restricted motion, often with fever (Figure 3C). Diagnosis is confirmed by synovial fluid aspiration and culture. MRI (Figure 3D) or ultrasound can detect effusion or osteomyelitis. Prompt empiric intravenous antibiotics covering Staphylococcus aureus (including MRSA) and gram-negative organisms, plus early surgical drainage, are critical to prevent cartilage destruction and permanent stiffness [29].

Osteomyelitis of the Distal Phalanx

Osteomyelitis is an infection of bone and surrounding soft tissue caused by pyogenic organisms introduced through trauma, bites, burns, or by hematogenous or contiguous spread [32, 33]. The distal phalanx of the dominant hand is most often involved. Patients present with erythema, swelling, dull pain, warmth, and tenderness, and may develop fever or malaise (Figure 3F,G). *Staphylococcus aureus* is the most common pathogen. Diagnosis is confirmed by bone biopsy and culture, while MRI provides the highest sensitivity for early detection [33]. Empiric intravenous antibiotics that cover gram-positive and anaerobic organisms should begin promptly and continue for four to six weeks, guided by culture results. Surgical debridement or resection is indicated when necrotic bone or an abscess is present. Delayed therapy increases the risk of chronic infection, deformity, or amputation [32].

Hand Infections Secondary to Uncontrolled Diabetes Mellitus

Diabetic hand infections encompass a wide range of disease processes, including cellulitis, abscess, paronychia, felon, septic arthritis, tenosynovitis, necrotizing fasciitis, and osteomyelitis, occurring most often in older adults with poorly controlled DM [34]. Hyperglycemia impairs leukocyte function and microvascular circulation, increasing susceptibility to infection and delayed healing [3]. The most common pathogens are *Staphylococcus* aureus, *Klebsiella *species, *Candida *species, and mixed bacterial or fungal organisms [34]. Patients commonly present with erythema, swelling, warmth, tenderness, and reduced mobility of the hand; severe infections may cause fever, tachycardia, or sepsis (Figure 3H,I). Diagnosis is clinical and is supported by cultures or imaging if deeper involvement is suspected. Management requires strict glycemic control, limb immobilization, surgical drainage when necessary, and parenteral antibiotics tailored to culture results. Delayed presentation, deep-space infection, or polymicrobial involvement increases the likelihood of amputation and functional loss [35].

Bite-related infections

Common mechanism-based infections with unique microbiologic profiles. Table 1 summarizes the common pathogens, empiric antibiotic choices, and unique management considerations for each bite type.

**Table 1:** Common pathogens and empiric management of bite-related hand infections MCP: metacarpophalangeal joint; IV: intravenous; spp.: species.

Bite Type	Common Pathogens	Empiric Antibiotic Therapy	Unique Management Considerations
Cat bite	*Pasteurella multocida*; *Staphylococcus aureus*; *Streptococcus *spp.; oral anaerobes	First line: oral amoxicillin clavulanate. Severe infection: IV ampicillin sulbactam. Severe beta-lactam allergy: clindamycin plus ciprofloxacin or levofloxacin; doxycycline plus metronidazole is another adult option, depending on local guidance.	High risk for deep puncture inoculation into the tendon sheath, joint, or bone. Avoid primary closure for most hand wounds. Consider imaging if a foreign body, deep-space infection, septic arthritis, or osteomyelitis is suspected.
Dog bite	*Pasteurella *spp.; *Staphylococcus aureus*; *Streptococcus *spp.; oral anaerobes; *Capnocytophaga canimorsus*	First line: oral amoxicillin clavulanate. Severe infection: IV ampicillin sulbactam. Severe beta-lactam allergy: clindamycin plus ciprofloxacin or levofloxacin; doxycycline plus metronidazole is another adult option, depending on local guidance.	Often causes crush injury with possible tendon, nerve, vascular, or bone involvement. Avoid primary closure for most hand wounds. Assess tetanus status and need for rabies prophylaxis.
Human bite	*Staphylococcus aureus*; viridans streptococci; *Eikenella corrodens*; oral anaerobes including *Fusobacterium*, *Prevotella*, and *Porphyromonas *spp.	First line: oral amoxicillin clavulanate. Severe infection: IV ampicillin sulbactam. Severe beta-lactam allergy: clindamycin plus ciprofloxacin or levofloxacin. Avoid clindamycin monotherapy because it lacks reliable Eikenella coverage.	A fight bite over the dorsal MCP joint may penetrate the extensor tendon or joint capsule. Requires careful irrigation, exploration, and often delayed closure. Assess tetanus status and consider hepatitis B, hepatitis C, and HIV risk based on exposure.

Cat Bite Infections

**Figure 4:**
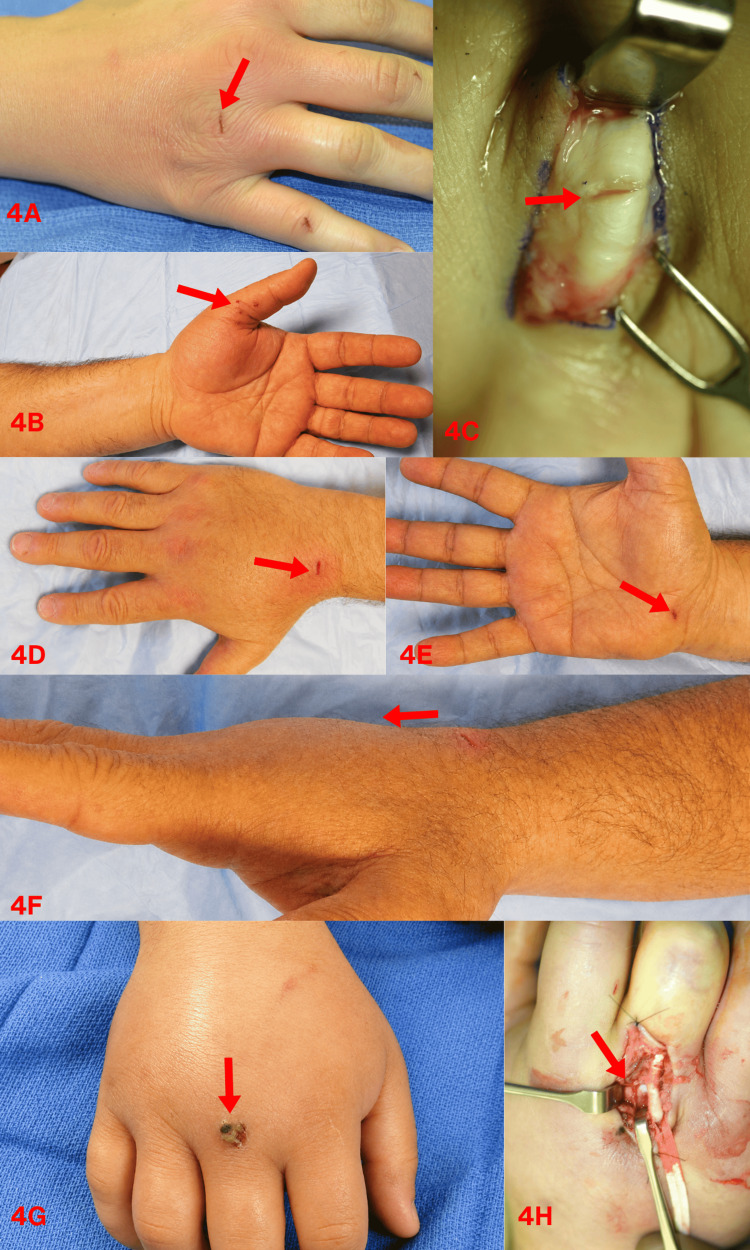
Bite-related infections (A-C) Cat bite injury: gross examination shows multiple puncture wounds on the dorsal (red arrow, A) and volar (red arrow, B) aspects of the hand; intraoperative examination (C) exhibits deep tissue disruption, with a sharp, elongated tooth mark (red arrow) causing an extensor tendon laceration of the right ring finger. (D-F) Dog bite injury, with erythema (red arrow, D), lacerations (red arrow, E), and swelling (red arrow, F) across the palm and dorsum of the hand, consistent with polymicrobial infection and early cellulitis with soft-tissue trauma. (G-H) Human fight bite injury: gross examination (G) shows a single laceration at the metacarpophalangeal joint of the right middle finger (red arrow); intraoperative findings (H) reveal a capsular laceration with partial disruption of the extensor mechanism (red arrow). Image courtesy of Gary Schwartz, MD. Used with permission.

The narrow teeth of cats create deep puncture wounds, allowing for direct inoculation of bacteria into poorly vascularized tissues such as tendon sheaths and joints [36]. Infections are usually polymicrobial, with *Pasteurella multocida *as the most common isolated pathogen [37]. Within 12 to 24 hours, symptoms include pain, swelling, erythema, and limited motion, particularly when the bite is over a joint or tendon sheath (Figure 4A-C). Diagnosis is clinical, supported by wound cultures and imaging when deep-space infection or foreign body retention is suspected [11]. Management includes thorough irrigation (Figure 4C), careful debridement, avoidance of primary closure, and empiric antibiotics effective against *Pasteurella *and mixed flora (e.g., oral amoxicillin-clavulanate) [38].

Dog Bite Infections

The broad, blunt teeth of dogs create crush and tearing wounds that can damage tendons, joints, and nerves, predisposing to deep-space infection [39, 40]. Within 12 to 48 hours, symptoms include erythema, swelling, pain, and occasionally fever (Figure 4D-F). Infections are often polymicrobial, involving *Pasteurella*, *Streptococcus*, and *Staphylococcus* species, along with anaerobes such as *Fusobacterium*, *Porphyromonas*, and *Bacteroides* [37]. Rabies virus remains a concern in endemic areas [40]. Diagnosis is clinical and supported by cultures; imaging may be warranted for suspected fracture, foreign body, or deep-space involvement. Management includes prompt irrigation, debridement, and empiric oral amoxicillin-clavulanate. Primary closure should be avoided for bite wounds to the hand, as the infection risk in this location is substantially higher than in other anatomical sites. Wounds to the face and other well-vascularized regions may tolerate primary closure when treated promptly and with appropriate antibiotics [39]. Rabies prophylaxis should be considered.

Human Bite Infections

Human bite wounds are prone to serious complications and are often polymicrobial, involving *Staphylococcus aureus*, *Streptococcus *species, *Eikenella corrodens*, and anaerobes such as *Fusobacterium *and *Bacteroides *[41,42]. Symptoms include pain, swelling, erythema, and purulent drainage (Figure 4G), typically within the first 24 hours of injury [40,41]. The most concerning mechanism is the fight bite, which occurs when a closed fist strikes the teeth of another person, producing a small laceration over the dorsal metacarpophalangeal joint that may extend into the joint capsule or tendon sheath (Figure 4H) [41]. Diagnosis is clinical, supported by wound cultures and imaging when evaluating for fractures, retained teeth, or deep involvement [40]. Management includes thorough irrigation, debridement, empiric amoxicillin-clavulanate, and delayed closure for high-risk wounds [41, 42]. Tetanus prophylaxis and viral prophylaxis should be considered. Early intervention prevents abscess, septic arthritis, and osteomyelitis [41].

Limitations

This retrospective pictorial review has several limitations. The cases were collected from the archive of a single board-certified hand surgeon, which may not represent the full spectrum of hand infection presentations encountered across different clinical settings or patient populations. Additionally, the retrospective nature of the case collection precludes standardized documentation and systematic follow-up data.

## Conclusions

Hand infections span a continuum from common superficial conditions to limb-threatening deep-space, articular, and osseous disease. Early recognition based on characteristic clinical clues, judicious use of imaging to detect occult abscess or joint involvement, and prompt initiation of empiric antibiotics with timely surgical consultation are essential to preserve function. The pictorial cases in this review highlight practical patterns that clinicians can use at the point of care to triage urgency, select initial management, and identify patients who require early referral or operative intervention. Consistent application of these principles can reduce complications, shorten recovery, and improve functional outcomes.

## References

[1] Osterman M, Draeger R, Stern P (2014). Acute hand infections. J Hand Surg Am.

[2] Kennedy CD, Huang JI, Hanel DP (2016). In brief: Kanavel's signs and pyogenic flexor tenosynovitis. Clin Orthop Relat Res.

[3] Malizos KN, Papadopoulou ZK, Ziogkou AN, Rigopoulos N, Athanaselis ED, Varitimidis SE, Dailiana ZC (2020). Infections of deep hand and wrist compartments. Microorganisms.

[4] Leggit JC (2017). Acute and chronic paronychia. Am Fam Physician.

[5] Bystritsky RJ (2021). Cellulitis. Infect Dis Clin North Am.

[6] Rockwell PG (2001). Acute and chronic paronychia. Am Fam Physician.

[7] Schwartz GB (1988). Subungual squamous cell carcinoma of a nailbed. A case report. Orthop Rev.

[8] Leung AK, Lam JM, Leong KF, Hon KL, Barankin B, Leung AA, Wong AH (2020). Onychomycosis: an updated review. Recent Pat Inflamm Allergy Drug Discov.

[9] Frazier WT, Santiago-Delgado ZM, Stupka KC 2nd (2021). Onychomycosis: rapid evidence review. Am Fam Physician.

[10] Westerberg DP, Voyack MJ (2013). Onychomycosis: current trends in diagnosis and treatment. Am Fam Physician.

[11] Lim SS, Ohn J, Mun JH (2021). Diagnosis of onychomycosis: from conventional techniques and dermoscopy to artificial intelligence. Front Med (Lausanne).

[12] Stevens DL, Bisno AL, Chambers HF (2014). Practice guidelines for the diagnosis and management of skin and soft tissue infections: 2014 update by the infectious diseases society of America. Clin Infect Dis.

[13] Jeng A, Beheshti M, Li J, Nathan R (2010). The role of beta-hemolytic streptococci in causing diffuse, nonculturable cellulitis: a prospective investigation. Medicine (Baltimore).

[14] Raff AB, Kroshinsky D (2016). Cellulitis: a review. JAMA.

[15] Griego RD, Rosen T, Orengo IF, Wolf JE (1995). Dog, cat, and human bites: a review. J Am Acad Dermatol.

[16] Liu C, Bayer A, Cosgrove SE (2011). Clinical practice guidelines by the infectious diseases society of america for the treatment of methicillin-resistant Staphylococcus aureus infections in adults and children. Clin Infect Dis.

[17] Al-Qattan MM, Murray KA, El-Shayeb A (2006). Arteriovenous vascular malformations confined to the hand: an algorithm of management based on a new classification. J Hand Surg Br.

[18] Dailiana ZH, Rigopoulos N, Varitimidis S, Hantes M, Bargiotas K, Malizos KN (2008). Purulent flexor tenosynovitis: factors influencing the functional outcome. J Hand Surg Eur Vol.

[19] Chapman T, Ilyas AM (2019). Pyogenic flexor tenosynovitis: evaluation and treatment strategies. J Hand Surg Am.

[20] Pang HN, Teoh LC, Yam AK, Lee JY, Puhaindran ME, Tan AB (2007). Factors affecting the prognosis of pyogenic flexor tenosynovitis. J Bone Joint Surg Am.

[21] Flevas DA, Syngouna S, Fandridis E, Tsiodras S, Mavrogenis AF (2019). Infections of the hand: an overview. EFORT Open Rev.

[22] Osipchuk D, Riddell J (2023). Bilateral infectious extensor tenosynovitis: a case report. Clin Pract Cases Emerg Med.

[23] Amini R, Camacho L, Acuña J, Situ-La Casse EH, Adhikari S (2020). Point of care ultrasound in pyogenic tenosynovitis: a case report. Bull Emerg Trauma.

[24] Plotkin B, Sampath SC, Sampath SC, Motamedi K (2016). MR imaging and US of the wrist tendons. Radiographics.

[25] Lipatov KV, Asatryan A, Melkonyan G, Kazantcev AD, Solov'eva EI, Cherkasov UE (2022). Septic arthritis of the hand: current issues of etiology, pathogenesis, diagnosis, treatment. World J Orthop.

[26] Meier R, Wirth T, Hahn F, Vögelin E, Sendi P (2017). Pyogenic arthritis of the fingers and the wrist: can we shorten antimicrobial treatment duration?. Open Forum Infect Dis.

[27] Kwak SH, Bae JY, Oh Y, Jang HS, Ahn TY, Lee SH (2020). Primarily treated patients versus referred patients in the treatment of native septic arthritis of digits: a retrospective comparative study. BMC Musculoskelet Disord.

[28] Sammer DM, Shin AY (2009). Comparison of arthroscopic and open treatment of septic arthritis of the wrist. J Bone Joint Surg Am.

[29] Goldenberg DL (1998). Septic arthritis. Lancet.

[30] Shirtliff ME, Mader JT (2002). Acute septic arthritis. Clin Microbiol Rev.

[31] Margaretten ME, Kohlwes J, Moore D, Bent S (2007). Does this adult patient have septic arthritis?. JAMA.

[32] Reilly KE, Linz JC, Stern PJ, Giza E, Wyrick JD (1997). Osteomyelitis of the tubular bones of the hand. J Hand Surg Am.

[33] Terrence Jose Jerome J, Kumar A, Chandar DM (2025). Surgical management for distal phalanx osteomyelitis: a narrative review. J Clin Orthop Trauma.

[34] Jalil A, Barlaan PI, Fung BK, Ip JW (2011). Hand infection in diabetic patients. Hand Surg.

[35] Raveendran S, Naik D, Raj Pallapati SC, Prakash JJ, Thomas BP, Thomas N (2016). The clinical and microbiological profile of the diabetic hand: a retrospective study from South India. Indian J Endocrinol Metab.

[36] Babovic N, Cayci C, Carlsen BT (2014). Cat bite infections of the hand: assessment of morbidity and predictors of severe infection. J Hand Surg Am.

[37] Talan DA, Citron DM, Abrahamian FM, Moran GJ, Goldstein EJ (1999). Bacteriologic analysis of infected dog and cat bites. Emergency Medicine Animal Bite Infection Study Group. N Engl J Med.

[38] Wangler S, Elias M, Schoepke L, Merky DN, Meier R, Vögelin E (2024). Cat bite injuries to the hand and forearm: the impact of antibiotic treatment on microbiological findings and clinical outcome. Arch Orthop Trauma Surg.

[39] Aziz H, Rhee P, Pandit V, Tang A, Gries L, Joseph B (2015). The current concepts in management of animal (dog, cat, snake, scorpion) and human bite wounds. J Trauma Acute Care Surg.

[40] Kennedy SA, Stoll LE, Lauder AS (2015). Human and other mammalian bite injuries of the hand: evaluation and management. J Am Acad Orthop Surg.

[41] Talan DA, Abrahamian FM, Moran GJ, Citron DM, Tan JO, Goldstein EJ (2003). Clinical presentation and bacteriologic analysis of infected human bites in patients presenting to emergency departments. Clin Infect Dis.

[42] Liliav B (2018). Human bites resulting in hand infections: is Eikenella a bug of the past?. Clin Surg.

